# Effect of high-intensity interval training in young heart transplant recipients: results from two randomized controlled trials

**DOI:** 10.1186/s13102-020-00180-1

**Published:** 2020-06-04

**Authors:** Kari Nytrøen, Katrine Rolid, Marianne Yardley, Lars Gullestad

**Affiliations:** 1 Department of Cardiology, Oslo University Hospital Rikshospitalet, postbox 4950, Nydalen, 0424 Oslo, Norway; 2 Faculty of Medicine, University of Oslo, Postbox 1072 Blindern, 0316 Oslo, Norway; 3 KG Jebsen Center for Cardiac Research, University of Oslo, Norway and Center for Heart Failure Research, Oslo University Hospital, Oslo, Norway

**Keywords:** Heart transplant, Young recipients, Rehabilitation, High-intensity interval training, Cardiopulmonary exercise test, Peak oxygen uptake, Peak oxygen consumption

## Abstract

**Background:**

Little is known about the effect of exercise in young heart transplant recipients, and results on group level is lacking. This study summarizes the findings of the youngest participants in two previous randomized controlled trials.

**Method:**

This is a hypothesis-generating study reporting the main results from the youngest participants in two larger randomized controlled trials investigating the effect of high-intensity interval training (HIT). The article summarizes the main results from 28 young participants (< 40 year of age) who have participated in two previous studies which evaluated the effect of HIT vs. controls in adult heart transplant recipients. One of the studies included de novo heart transplant recipients and the other included maintenance heart transplant recipients.

All study tests were performed in-hospital, in the specialist health care setting, but the exercise intervention was carried out locally, in cooperation with the primary health care. In both studies the exercise intervention lasted for 9–12 months. In one study, HIT (85–95% of peak effort) was compared to controls (no specific intervention), and in the other study HIT was compared to moderate, continuous exercise (MICT*,* 60–80% of peak effort). The main outcome measure was peak oxygen uptake (VO_2peak_) and a secondary endpoint was muscle strength.

**Results:**

The summarized findings from the youngest heart transplant recipients in these two studies demonstrated mainly that the improvement in peak oxygen uptake among the younger recipients (< 40 years) was much larger (4.7 vs. 1.2 ml/kg/min and 7.0 vs. 2.2 ml/kg/min) compared to the improvement among the older recipients (≥ 40 years), and in accordance with results from adult heart transplant populations: HIT, compared to MICT, induced the largest improvement in peak oxygen consumption, also in the younger heart transplant recipients.

**Conclusions:**

These results suggest that young heart transplant recipients have a greater effect of HIT than of MICT and may also suggest that younger recipients benefit more from high-intensity interval training than their older co-patients. However, larger randomized studies focusing on the young heart transplant population is strongly needed to confirm this hypothesis.

**Trial registration:**

Clinical trial registrations: NCT01796379 and NCT01091194.

## Background

Little is known about the effect of exercise in young heart transplant recipients, but most of the few studies that exist report benefits in overall exercise capacity as well as improved health-related quality of life [1–5]. Furthermore, the literature demonstrates individuals’ participation and excellent achievements in national and international transplant games; in competitive cycling, in grueling endurance competitions as the Ironman, and in climbing the world’s tallest peaks [6]. Yet, results on group level is lacking, and more research in this area is highly warranted.

Although survival is significantly higher in pediatric heart transplant recipients than in adult heart transplant recipients: conditional pediatric median survival is 21 years vs. 13 years in adults [7], this has a potential to be further improved. It is recently shown that measures of physical capacity are highly associated with survival in adult heart transplant recipients [8] and thus, it is likely to believe that this is true also for the younger population.

Our research group has, to date, conducted the two largest randomized controlled trials that exist on the effect of high-intensity interval training in adult heart transplant recipients: the HITTS study (High-intensity Interval Training in de novo heart Transplant recipients in Scandinavia), [9, 10], and the TEX (Transplant EXercise) study among maintenance heart transplant recipients [11]. The purpose of the current article is to highlight and report the effect of high-intensity interval training (HIT) vs. moderate intensity, continuous training (MICT) or no training among the youngest heart transplant recipients. This is a hypothesis-generating study only, reporting the main results from the younger participants (< 40 years of age) (*n* = 28) in these two studies. The initial plan was to evaluate the recipients < 30 years of age, but due to a very limited number of young participants in the two trials the cut-off was extended to 40 years. Results from the older group of patients (≥ 40 years of age) are also reported as supplementary material (Table 3), for numerical comparisons only.

## Methods

### The HITTS study (High-intensity interval training in de novo heart transplant recipients in Scandinavia)

The most recent randomized controlled trial (ClinicalTrial.gov registration: NCT01796379) started its inclusion in 2013, and 81 de novo heart transplant patients > 18 years of age were included 8–12 weeks post heart transplant. The 1-year follow-up was completed by the end of 2017, and the 3-year follow-up was completed by the end of 2019. The patients were randomized to either 9 months of high-intensity interval training or 9 months of moderate intensity, continuous training. Further details about the study is published in a design-paper [9], and comprehensive results from the 1-year follow-up was recently published [10]. Of the 81 included patients in the main study, 78 patients completed the 1-year follow-up and of these, 16 patients (20.5%) were < 40 years of age, with a mean ± age of 28.3 ± 6.5 years (Table 1).

**Table 1:** Baseline characteristics of the two study populations < 40 years of age

	The HITTS study (***n*** = 16)		The TEX study (***n*** = 12)	
	HIT group (***n*** = 6) Mean ± SD or median (IR)	MICT group (***n*** = 10) Mean ± SD or median (IR)	HIT group (***n*** = 8) Mean ± SD or median (IR)	Control group (***n*** = 4) Mean ± SD or median (IR)
Age (years)				
Range 18–39 in both studies	29.1 ± 7.6	27.8 ± 6.2	27.1 ± 7.6	28.0 ± 7.8
Gender (count)	Women: 0, Men:6	Women:5, Men:5	Women: 3, Men: 5	Women: 2, Men: 2
Time after heart transplant at inclusion	11 ± 2.7 (weeks)	11 ± 1.5 (weeks)	4.4 ± 3.3 (years)	3.0 ± 0.0 (years)
Waitinglist (days)	70 (160)	62 (138)		
Donor age (years)	31 (35)	31 (23)	33 (17)	38 (29)
Ischemic time (min)	209 (206)	214 (112)	225 (45) †	70 (95) †
Creatinine (μmol/l)	115 (35)	105 (60)	91 (28) †	77 (16) †
Primary diagnosis (count)				
Cardiomyopathy	3	9	7	3
Coronary artery disease	1	0	0	0
Other	2	1	1	1

† *p-value < 0.05 between groups at baseline (Mann-Whitney U -test). There were no other baseline differences between the exercise-groups*

*SD* Standard deviation, *IR* Interquartile range, *HIT* High-intensity interval training, *MICT* Moderate intensity continuous training, *HITTS* High-intensity Interval Training in de novo heart Transplant recipients in Scandinavia, *TEX* Transplant EXercise

### The HITTS intervention

**Fig. 1:**
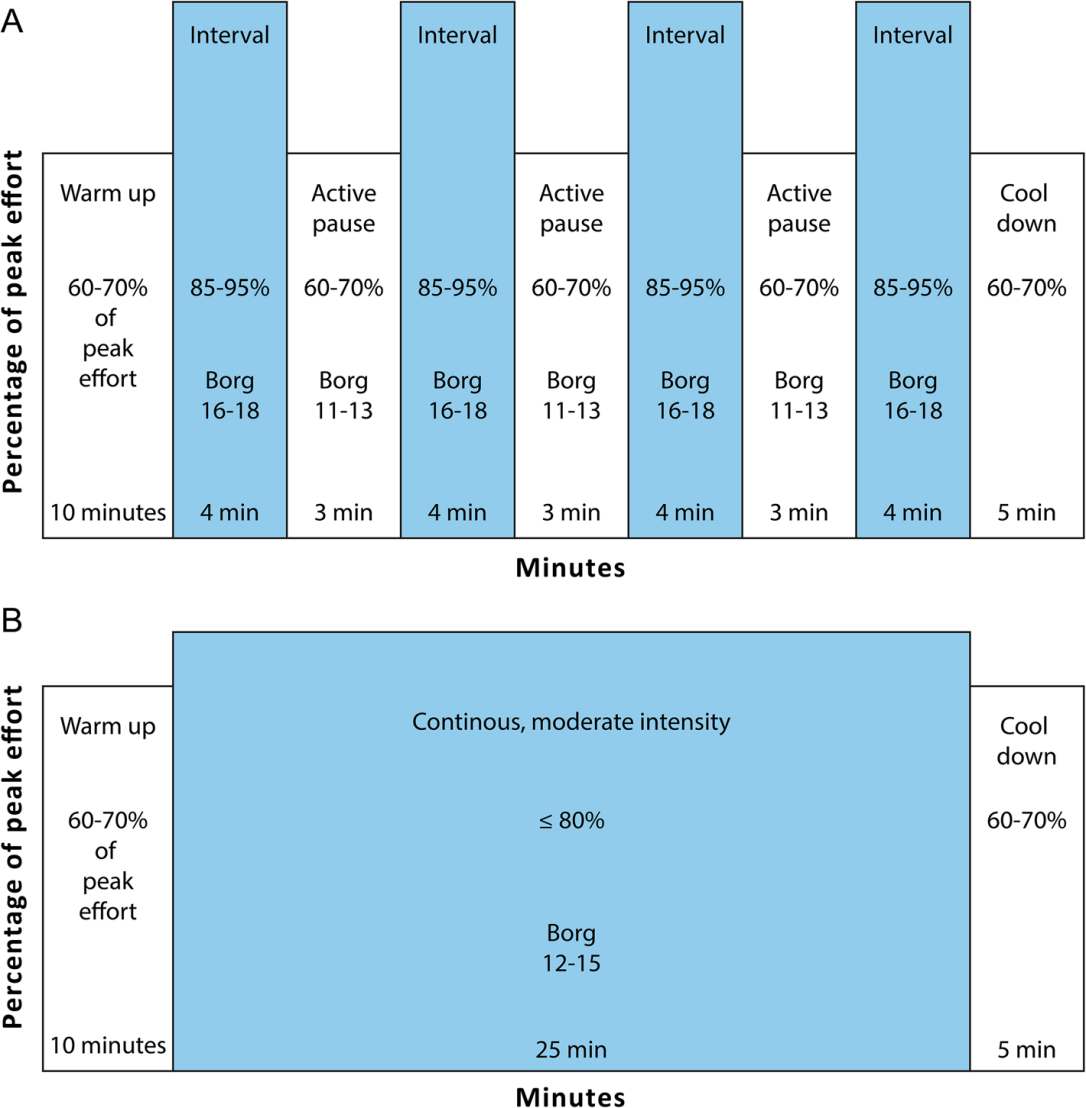
Illustration of the two exercise protocols: a session of (**a**) high-intensity interval training (HIT) and (**b**) moderate intensity continuous training (MICT). Legend: (This figure has previously been published in Am Heart J 2016;172:96–105. Reproduced with permission. https://www.sciencedirect.com/science/article/pii/S0002870315006286)

All the included patients started supervised exercise in their home communities, after discharge from the hospital, approximately 3 months post transplantation. The high-intensity interval training group performed exercise on a treadmill at an intensity between 85 and 95% of peak effort (Fig. 1a). The intervention was conducted locally, in each patient’s home community and every single exercise session was supervised and closely monitored by a physical therapist. The moderate intensity training group performed “traditional” exercise with continuous intensity between 60 and 80% of peak effort (Fig. 1b). Both groups performed the same amount of sessions throughout the 9-month long intervention period. At the 1-year follow-up the primary outcome measure was peak oxygen uptake measured from a cardiopulmonary exercise test performed on a treadmill. Important secondary outcome variables were muscular endurance and maximum muscle strength. Further details about the intervention and the measurements are previously published [9, 10].

### The TEX study (transplant EXercise)

This randomized controlled trial (ClinicalTrial.gov registration: NCT01091194), conducted in 2009–2010 included 52 heart transplant patients > 18 years of age, 1–8 years after heart transplant, and 48 of these completed the 1-year follow-up. The patients were randomized to either 1 year of supervised high-intensity interval training or a control group which continued as before with their regular activities. Further details about the population and the study design are previously reported [11, 12]. Of the 48 patients who completed follow-up, 12 patients (25%) were < 40 years of age with a mean ± age of 27.4 ± 7.3 years (Table 1).

### The TEX intervention

The intervention was conducted locally, in each patient’s home community and consisted of high-intensity interval training performed on a treadmill at an intensity between 85 and 95% of peak effort (Fig. 1a). Every single exercise session was supervised and closely monitored by a physical therapist. The 1 year of intervention was divided into three 8-week periods with three sessions/week; a total of 72 planned supervised sessions. In between these periods, self-exercise was strongly encouraged. The primary outcome measure was peak oxygen uptake from a cardiopulmonary exercise test performed on a treadmill. Important secondary outcome variables were muscular endurance and maximum muscle strength. Further details about the intervention and the measurements are previously published [11]. The control group continued as before with their everyday activities, but without any specific exercise intervention.

The HIT protocol in the two randomized studies was chosen because this is a well-known protocol, easy to adapt to a clinical setting and has been frequently used in other study populations with good results [13].

### Main outcomes

In the current sub-study (young heart transplant recipients), we only focused on the two main physical capacity measurements from the two included trials: peak oxygen uptake (ml/kg/min) measured during a cardiopulmonary exercise test performed on a treadmill, and isokinetic testing of muscle strength: both muscular exercise capacity (Joules) and maximum muscle strength (Newton meters) measured during knee-extension [11]. Additionally, heart rate variables and chronotropic response are reported because these variables are closely related to peak oxygen consumption and the heart transplant patients’ denervated heart, especially the newly transplanted patients have an attenuated heart rate response (the HITTS study).

### Statistical analysis

Continuous data are expressed as mean ± standard deviation (SD) or median (interquartile range (IR)), and categorical data are presented as counts/percentages. Although the sample is small (age < 40 years) we performed between-group comparisons using unpaired t-tests of the mean change. In cases of skewed distribution, non-parametric Mann Whitney U tests were also performed. The between-group analyses were performed between the youngest participants in the two randomized controlled to explore whether young heart transplant patients seem to benefit from HIT, similarly to populations with a higher mean age. Thus, the statistics must be interpreted with caution and it must be underscored that this is a hypothesis-generating study. A supplemental table (Table 3) with the results from the older participants is provided for numerical comparisons for those interested. Because of the large difference in group-sizes, statistics could not be performed to compare the young vs. the old, which was not the main scope of this study.

## Results

### Clinical characteristics

The baseline characteristics of the patients in the two study populations (*n* = 28) are presented separately in Table 1. In both populations, the mean age was 27–28 years (range 18–39) (mean ± SD: 28.3 ± 6.5/ 27.4 ± 7.3) and the majority were men. In the HITTS study (*n* = 16), the patients were baseline-tested at mean ± SD 11 ± 3 weeks post heart transplant and baseline mean ± SD peak oxygen consumption was 23.3 ± 5.7 ml/kg/min. In the TEX study (*n* = 12), baseline mean ± SD time after heart transplant was 4.0 ± 2.1 years (range 1–8), and baseline mean ± SD peak oxygen consumption was 29.0 ± 6.3 ml/kg/min. The baseline peak oxygen consumption values were not significantly different between groups in either of the studies. Potential confounders between the age-matched exercise-groups could be primary diagnosis, ischemic time, donor age and creatinine level. These variables are presented in Table 1. In this small, hypothesis-generating sub-study looking into several other possible confounders would only be of speculative nature.

### Physical capacity

**Fig. 2:**
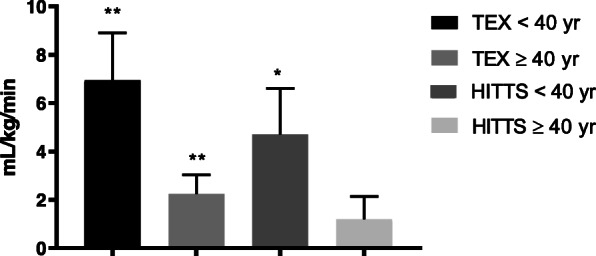
Mean change ± SE in VO2 peak between exercise groups in both trials at follow-up. Legend: A visualization of young vs. old in the TEX and the HITTS study. The bars marked with “**” or “*” means that the mean change was significant (*p* < 0.01, or *p* < 0.05) at follow-up, as presented in Tables 2 and 3

Figure 2 visually illustrates the mean change at follow-up between the different exercise groups in the different studies given in Table 1 for the youngest participants and in Table 3 for the older participants. During the 9–12 months of exercise training in both studies, the mean change in peak oxygen consumption between the exercise groups, seems to be greater in patients < 40 years of age compared to the patients ≥40 years of age, and this appears mainly to be driven by a greater effect of the high-intensity interval training in the youngest participants (Table 1).

### Young recipients in the HITTS-study

The high-intensity interval training group had a significantly higher improvement in peak oxygen consumption at 1-year follow-up compared to the moderate intensity training group. The mean [95% CI] change between groups in peak oxygen consumption was 4.7 [0.6, 8.8] ml/kg/min (*p* = 0.028) (Table 2, Fig. 2).

**Table 2:** Effect of exercise in the two study populations < 40 years of age

	The HITTS study (*n* = 16)						The TEX study (*n* = 12)					
	HIT group (***n*** = 6) Mean ± SD or median (IR)		MICT group (***n*** = 10) Mean ± SD or median (IR)		Mean difference between groups [95%CI]	*t*-test *p*-value	HIT group (***n*** = 8) Mean ± SD		Control group (***n*** = 4) Mean ± SD		Mean difference between groups [95%CI]	*t*-test *p*-value
	Baseline	Follow-up	Baseline	Follow-up			Baseline	Follow-up	Baseline	Follow-up		
VO_2peak (ml/kg/min)_	22.3 ± 5.6	30.0 ± 8.9	24.0 ± 6.0	27.0 ± 7.7	4.7 [0.6, 8.8]	**0.028**	27.0 ± 4.7	32.5 ± 4.5	33.0 ± 8.0	31.5 ± 5.1	7.0 [2.6, 11.3]	**0.005**
%VO_2exp_	51.3 ± 12.9	69.9 ± 20.4	59.7 ± 13.6	67.5 ± 16.5	11 [1, 20]	**0.030**	66.3 ± 9.6	81.2 ± 13.3	82.5 ± 17.6	79.4 ± 10.3,	18.0 [5.9, 30.0]	**0.008**
O_2_ pulse	13.0 ± 3.0	15.4 ± 3.3	12.0 ± 2.6	11.7 ± 2.9	2.7 [− 0.1, 5.6]	0.060	15.1 ± 3.7	16.3 ± 2.8	16.7 ± 5.3	16.6 ± 4.3	1.4 [− 0.5, 3.2]	0.132
HR rest echo	91 ± 8	91 ± 10	83 ± 7	91 ± 8	8 [0, 17]	0.053	85 ± 16	80 ± 12	75 ± 12	84 ± 17	− 14 [− 22, − 6]	**0.003**
HR peak	127 (29)	161 (43)	136 (39)	166 (30)		0.064†	156 ± 15	165 ± 17	163 ± 16	165 ± 18	7 [− 8, 15]	0.073
%HR max	68 ± 6	81 ± 8	69 ± 11	84 ± 11	1 [− 7, 9]	0.731	81 ± 7	86 ± 8	85 ± 11	87 ± 10	4 [− 1, 8]	0.085
Chronotropic response index	0.38 ± 0.12	0.63 ± 0.20	0.47 ± 0.17	0.70 ± 0.24	0.02 [− 0.15, 0.19]	0.770	0.67 ± 0.11	0.77 ± 0.12	0.77 ± 0.14	0.77 ± 0.16	0.09 [0.01, 0.17]	**0.031**
HR recovery 2 min (bpm)	−2 ± 3	−14 ± 12	−3 ± 2	− 23 ± 10	−9 [− 21, 3]	0.141	− 22 ± 5	−30 ± 6	− 39 ± 5	− 38 ± 10	− 8 [− 17, 0]	0.069
Quadriceps muscular exercise capacity (J)	2944 (3017)	3284 (2961)	1649 (1774)	2524 (2824)		0.162†	2813 ± 2042	3572 ± 1598,	3605 ± 1675	3697 ± 1138	667 [− 382, 1716]	0.187
Quadriceps maximum strength (Nm)	216 (124)	288 (136)	172 (143)	201 (175)		**0.006**†	240 ± 117	264 ± 111	279 ± 90	262 ± 83	41 [5, 77]	**0.031**
BMI	23.5 ± 4.4	24.6 ± 4.9	23.2 ± 4.4	24.1 ± 4.9	0.2 [− 1.9, 2.2]	0.858	27.4 ± 5.1	25.8 ± 4.6	25.7 ± 3.5	25.4 ± 2.3	− 1.2 [− 4.4, 1.9]	0.410

† *Mann Whitney U-test*

*SD* Standard deviation, *IR* Interquartile range, *HITTS* High-intensity Interval Training in heart Transplant recipients in Scandinavia, *TEX* Transplant EXercise, *HIT* High-intensity interval training, *MICT* Moderate intensity continuous training; %VO_2exp,_ percentage of expected VO_2 peak_ level according to age; *HR* Heart rate; %HR max, percentage of maximum HR according to age; *J* Joule, *Nm* Newton meter

In comparison, *n* = 62 patients ≥40 years of age (mean ± SD) age 54 ± 8, range 40–69), the mean [95%CI] difference in peak oxygen consumption between the high-intensity interval training group and the moderate, continuous training group was much smaller: 1.2 [− 0.7, 3.1] ml/kg/min (*p* = 0.215) (Table 3, Fig. 2).

**Table 3:** Effect of exercise in the two study populations ≥40 years of age

	The HITTS study (*n* = 62)						The TEX study (*n* = 35)					
	HIT group (***n*** = 31) Mean ± SD		MICT group (***n*** = 31) Mean ± SD		Mean difference between groups [95%CI]	*t*-test *p*-value	HIT group (***n*** = 16) Mean ± SD		Control group (***n*** = 19) Mean ± SD		Mean difference between groups [95%CI]	*t*-test *p*-value
	Baseline	Follow-up	Baseline	Follow-up			Baseline	Follow-up	Baseline	Follow-up		
Gender (count)	Women:9, Men: 22		Women:7, Men: 24				Women:5, men:11		Women:4, men; 15			
Time after heart transplant at inclusion	11 ± 1.8 (weeks)		11.2 ± 1.8 (weeks)				4.3 ± 2.4 (years)		4.1 ± 2.3 (years)			
VO_2peak_ (ml/kg/min)	19.0 ± 3.9	23.3 ± 5.5	20.4 ± 4.8	23.5 ± 6.2	1.2 [− 0.7, 3.1]	0.215	28.0 ± 6.0	30.0 ± 5.6	28.3 ± 6.0	28.1 ± 6.2	2.2 [0.6, 3.8]	**0.008**
%VO_2exp_	53.7 ± 11.5	66.0 ± 14.7	58.0 ± 12.4	66.7 ± 14.5	3.5 [− 1.8, 8.7]	0.190	86.6 ± 20.5	92.8 ± 18.5	84.2 ± 19.4	84.4 ± 19.4	6.1 [0.5, 11.6]	**0.033**
Quadriceps muscular exercise capacity (J)	2072 ± 816	2997 ± 1161	2560 ± 1133	2986 ± 1067	499 [65, 932]	**0.025**	3070 ± 1183	3383 ± 1058	2840 ± 781	3022 ± 937	131 [− 193, 456]	0.416
Quadriceps maximum strength (Nm)	179 ± 74	228 ± 80	195 ± 63	232 ± 65	12 [− 14, 38]	0.363	267 ± 72	259 ± 72	237 ± 68	225 ± 59	4 [−15, 22]	0.690

*SD* Standard deviation, *HITTS* High-intensity Interval Training in heart Transplant recipients in Scandinavia, *TEX,* Transplant EXercise; HIT, high-intensity interval training; MICT, moderate intensity continuous training; %VO_2exp,_ percentage of expected VO_2 peak_ level according to age, *J* Joule, *Nm* Newton meter

**Fig. 3:**
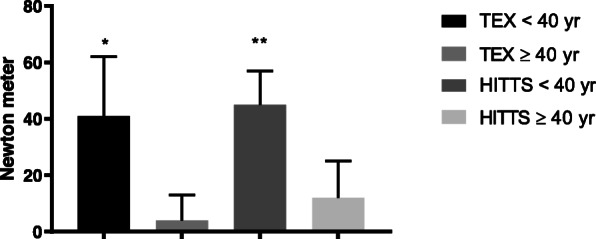
Mean change ± SE in extensors’ maximum strength between exercise-groups in both trials at follow-up. Legend: A visualization of young vs. old in the TEX and the HITTS study. The bars marked with “**” or “*” means that the mean change was significant (*p* < 0.01, or *p* < 0.05) at follow-up, as presented in Tables 2 and 3

**Fig. 4:**
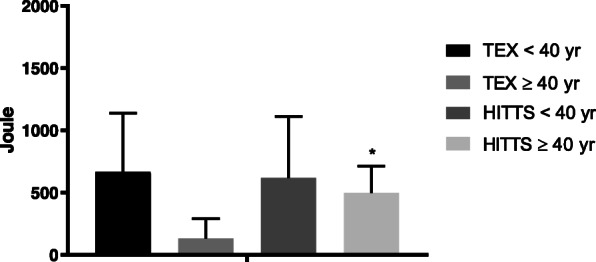
Mean change ± SE in extensors’ muscular exercise capacity between exercise-groups in both trials at follow-up. Legend: A visualization of young vs. old in the TEX and the HITTS study. The bars marked with “**” or “*” means that the mean change was significant (*p* < 0.01, or *p* < 0.05) at follow-up, as presented in Tables 2 and 3

The young high-intensity interval training group also demonstrated a higher maximum muscle strength than the young moderate, continuous training group, with a significant mean [95% CI] change between groups at 1-year follow-up of 45 [18,80] Newton meters (*p* = 0.004), while improvement in muscular exercise capacity (Joule) was similar in both groups (Table 2, Figs. 3 and 4).

Detailed results from the study population as a whole can be read in the main publication from the HITTS study [10].

### Young recipients in the TEX-study

In favor of the high-intensity interval training group the mean [95% CI] change in peak oxygen consumption between groups was 7.0 [2.6, 11.3] ml/kg/min (*p* = 0.005) at follow-up (Table 2) also in this study. Maximum muscle strength was also significantly higher in the high-intensity interval training group compared to the control group (no exercise group) at follow-up, with a mean [95% CI] difference of 41 [5, 77] Newton meters (*p* = 0.031) (Table 2, Fig. 3), while the difference in muscular exercise capacity was non-significant (Table 2, Fig. 4). Additionally, the high-intensity interval training group improved their resting heart rate and chronotropic response index more than the control group (*p* < 0.05) (Table 2).

Compared with the *n* = 35 older participants ≥40 years of age (mean ± SD age 59 ± 8), range 42–71), the mean [95%CI] difference in peak oxygen consumption between the high-intensity interval training group and the control group (no-exercise group) was much smaller at follow-up: 2.2 [0.6, 3.8 ml/kg/min (*p* = 0.008) (Table 3, Fig. 2). Detailed results from the study population as a whole can be read in the main publication from the TEX study [11].

## Discussion

The findings in the current study demonstrated mainly two things: 1) Among the young heart transplant recipients, high-intensity interval training induced the largest improvement in peak oxygen consumption, which is in accordance with results from adult heart transplant populations. 2) The improvement among the young recipients (< 40 years) seems to be much larger compared to the improvement among the older recipients (≥ 40 years).

Although there are only 28 young participants in these two studies, the results may suggest that high-intensity interval training is superior to moderate intensity, continuous training also among the young recipients and that the young may benefit more from high-intensity interval training than older recipients, especially in the de novo state. The current findings draw the attention towards the great potential systematic high-intensity interval training may have among the young heart transplant population, and also that future research maybe could differentiate and evaluate exercise interventions according to age.

What induces the “high-intensity interval training effect” is still unclear. In the adult maintenance heart transplant population, the improved peak oxygen consumption seems to rely on mostly peripheral changes such as improved muscle strength and function [11, 14–17]. In the de novo heart transplant population, the peak oxygen consumption improvement during the first year is more complex to describe, but seems to be associated with both central and peripheral factors [10]. This is also reflected in the current young HITTS population where the high-intensity interval training group had a borderline significantly higher O_2_ pulse (Table 2), which again by some is associated with a higher stroke volume [18].

Today, formal exercise programs are routine at the majority of adult heart transplant centers, and despite the lack of a clear consensus of what type/frequency/intensity of exercise that gives the most optimal results [17, 19], the answer to the question whether exercise is good for the heart transplant recipient is unequivocally yes [20]. Scientific evidence in this field is accumulating and most adults are at least offered some form of rehabilitation program after a heart transplant. So far none of the pediatric studies demonstrate such practice for the young heart transplant recipients. The accumulating evidence showing that HIT is a feasible, safe and effective form of exercise suggest that this should be used among a broader audience [20]. The current study demonstrated that HIT seems to be superior to MICT also among the younger HTx population but this warrants future and larger studies for the hypothesis to be confirmed.

The largest report to date, published in 2017, describes the functional status of > 1500 US children with a heart transplant [21]. This report is uplifting and states that > 60% have an excellent functional status (i.e. “normal and fully active”). Factors associated with a lower functional status were older age at the time of heart transplant, early rejections, African American race, hospitalization status at the time of heart transplant, a higher level of cardiac support at the time of heart transplant, and being on chronic steroids at the time of heart transplant [21]. An older study from 2006 has reported that exercise performance in 28 pediatric heart transplant recipients were impaired and declined over time in all the subjects [22], and baseline assessment from a new, ongoing study states that 13 heart transplant patients (mean age 15 years) have abnormal cardiac, vascular, and functional health indices, poor dietary habits, and are sedentary [23]. Knowing that some young heart transplant patients do have smaller or greater demands regarding keeping up with their peers in school, sports, higher education and career, a greater focus and effort in improving their physical capacity and correlated health related quality of life is needed [1].

The greatest limitation in this study is the small sample size drawn from two different studies and thus, the results must be interpreted with great caution. Significant differences between groups in the two main studies [10, 11], which turned out not to be significant among the younger recipients are likely to be due to type 2 errors. Furthermore, the included subjects are the youngest proportion of an adult study population and is not representative for children/teenagers. However, given the scarce documentation in this field we believe the results from the current study add interesting information to the discussion and contribute to generate new hypotheses for future research.

## Conclusion

In conclusion, physical rehabilitation should be required for all young heart transplant recipients regardless of functional status. The few studies that exist on effect of exercise in pediatric and young heart transplant patients report benefits in overall exercise capacity as well as improved health related quality of life, and it is reasonable to think that the accumulating evidence of the positive effects of high-intensity interval training in adult recipients is transferable to the younger recipients. Maybe the younger recipients benefit even more from high-intensity interval training than their older co-patients. However, larger randomized studies, especially among the young heart transplant population is strongly needed to confirm this hypothesis.

## Data Availability

The datasets generated and analyzed during the current study are not publicly available due to strict and limited data sharing possibilities as set by the South-East Regional Committee for Medical and Health Research Ethics in Norway. With reference to the European General Data Protection Regulation (GDPR), the data are personal data and thereby protected by secrecy.

## Abbreviations

CI: Confidence interval
HIT: High-intensity interval training
IR: Interquartile range
MICT: Moderate intensity continuous training (60–80% of peak effort)
SD: Standard deviation
The HITTS study: Effect of High-intensity Interval Training in de novo heart Transplant recipients in Scandinavia
The TEX study: The Transplant EXercise study
VO_2peak_: Peak oxygen consumption
